# Midlife Vulnerability and Food Insecurity in Women: Increased Risk of Mental Health Concerns

**DOI:** 10.3390/nu17152486

**Published:** 2025-07-30

**Authors:** Lisa Smith Kilpela, Taylur Loera, Sabrina E. Cuauro, Carolyn Black Becker

**Affiliations:** 1 Center for Research to Advance Community Health (ReACH Center), Department of Medicine, Lozano-Long School of Medicine, University of Texas Health Science Center at San Antonio, San Antonio, TX 78229, USA; loerat@uthscsa.edu; 2 Department of Psychological Sciences, Rice University, Houston, TX 77005, USA; sc235@rice.edu; 3 Department of Psychology, Trinity University, San Antonio, TX 78212, USA; cbecker@trinity.edu

**Keywords:** food insecurity, mental health, women’s health, developmental life stages

## Abstract

Background/Objectives: A growing body of literature has demonstrated that living with food insecurity (FI) increases risk for mental health concerns in addition to nutritional deficits (e.g., suboptimal micronutrient consumption, excessive macronutrient consumption, malnutrition). Yet, research is needed to improve our understanding of subpopulations potentially at increased risk for mental health concerns when living in the context of FI. The current study examined psychosocial health across women of different developmental life stages all living with FI. Methods: Female clients of a large, urban food bank (*N* = 680) living with FI completed measures of mental health and health-related quality of life (HRQOL) in a cross-sectional design conducted on site at the food bank. Results: Consistent with past research, FI severity was correlated with poorer psychosocial health across all variables. A multivariate analysis of covariance compared women living with FI across 4 developmental life stages (young adult, early midlife, late midlife, and older adult; age range = 18–94 years), controlling for FI severity and race/ethnicity, on outcomes related to mental health and HRQOL. Women in early and late midlife reported higher anxiety, eating disorder symptoms, and eating-related psychosocial impairment than younger and older women. Conclusions: The mental health toll of living with FI is profound; midlife may comprise a developmental period of increased vulnerability to experience this mental health burden of living with FI for women. Thus, efforts are needed to develop innovative pathways for interventions to support the mental health of midlife women living with FI, likely involving multi-level and/or multicomponent approaches to resource access.

## 1. Introduction

Food insecurity is a term used to describe the condition of being unable to afford and access food of sufficient quantity, quality, and variety [1,2]. Importantly, living with food insecurity is linked with poorer health and overall nutritional status, including suboptimal micronutrient consumption, excessive macronutrient consumption, and malnutrition. An estimated 13.5% of United States households experience food insecurity [3]. Food insecurity occurs across the adult lifespan in the United States (US), though research suggests that food insecurity is lower in older adults as compared to working age adults and younger adults [4]. The most common explanation for this finding is that the social safety net (i.e., Social Security) confers some protection for older adults [5]. However, because food insecurity is associated with worsened diet quality, it also increases the risk for (a) age-related health concerns including diabetes, hypertension, cardiovascular disease, decreased muscle mass, frailty and sarcopenic obesity [4,6,7,8] and (b) mortality [9]. Thus, a shorter lifespan may also play a role in the lower prevalence observed in older adults [4]. With regards to other differences in food insecurity trends for adults, research is somewhat inconsistent. However, a growing body of research suggests that both food insecurity and chronic conditions that can impact and result from food insecurity may disproportionately impact adults in midlife [4]. Moreover, midlife food insecurity may worsen health outcomes later in life. For instance, recent research indicates that midlife food insecurity appears to worsen cognitive functioning later in life [10].

There are currently no accepted definitive age cutoffs for midlife; it is often thought of as a life stage in which one is no longer young but not yet old that is accompanied by certain developmental tasks [11,12]. However, generally midlife is seen as starting somewhere between 40 and 45 and ending between 60 and 65 [4,12,13]. Midlife presents several challenges. First, it is the age in which many chronic diseases onset; it also is associated with the onset of functional impairment associated with aging [4,12,14]. Importantly, health deterioration in midlife appears to particularly impact women [14]. Indeed, research indicates that bone loss, negative alterations in lipid profiles, diabetes, and sleep difficulties all start or worsen during midlife in women; some of these changes are a result of the menopausal transition although others appear to simply occur concurrently [14]. Further, Karvonen-Gutierrez et al. (2013) found that two thirds of midlife women in early post-menopause showed indicators of osteoarthritis, which is a leading cause of pain and disability in adults in the US [15]. This age range also is a critical vulnerability window for risk of cardiovascular disease in women [14].

In addition to health and functionality-related difficulties, midlife poses a number of other challenges that affect women disproportionately [16]. For instance, many women in this life stage find themselves “sandwiched” with the need to simultaneously provide care to both children and parents while also managing a household and increasing workplace responsibilities associated with increasing seniority at work [16]. Within the theoretical literature, the term role overload is used to describe the pressure to manage too many role demands given available resources (e.g., time) [17]. Role overload can make midlife a particularly stressful stage of life for women. Further, role overload particularly when combined with the above-mentioned physical health challenges, the menopausal transition, and other life changes associated with midlife (e.g., divorce/breaking up with partner; death of parents; [18]), may also contribute to worsened mental health. Extant research supports the contention that midlife women are at elevated risk of depression. Specifically, research indicates that women are more likely to experience depression than men and are more likely to experience it during peri- or post-menopause relative to pre-menopause [19]. Further, research suggests that role overload is associated with poorer mental health in adult women [20].

It is important to note that financial disadvantage generally and food insecurity specifically both serve as additional stressors for midlife women of lower socioeconomic status. Further, a recent scoping review found support for a bidirectional relationship between food insecurity and depression (with each worsening the other) in women [21]. Despite this, no research to our knowledge has investigated the mental health of midlife women who live with food insecurity relative to other adult women who report food insecurity. The aim of this study was to conduct a secondary analysis of an existing dataset to examine self-reported mental health (depression, anxiety, eating disorder symptoms) and health-related quality of life (HRQOL) in midlife women as compared to younger and older adult women. All women were living with food insecurity. Based on the above literature, we hypothesized that midlife women would report greater depression, anxiety, eating disorder symptoms, and eating-related psychosocial impairment relative to both younger and older women. We also hypothesized that midlife women would report worsened HRQOL.

## 2. Materials and Methods

### 2.1. Participants

Participants were women (*N* = 680) living with food insecurity (FI) and other nonmedical determinants of health (NMDoH) who presented to the San Antonio Food Bank (SAFB) for neighbor services (*M*age = 47.7 years, SD = 16.1; range 18–94 years). Of note, we use the updated terminology within the hunger community, as well as the culture and community of our local food bank, of “neighbor” versus “client” to represent individuals receiving services at the food bank (i.e., the food bank serves their neighbors). Most participants (79.4%) self-identified their ethnicity as Hispanic/Latina. Regarding education, 33.4% reported less than a high school diploma/GED, and 37.2% reported obtaining either a high school diploma or GED. Most neighbors (63.5%) reported an annual household income of ≤$12,000, and 55.6% reported having at least one minor living in the home. Regarding employment status, 20.6% reported disability status. See Table 1 for a breakdown of sociodemographics by group.

### 2.2. Procedure

This study was conducted in collaboration with the San Antonio Food Bank (SAFB), with Institutional Review Board (IRB) approval by Trinity University. Texas ranks 2nd in the nation for FI, with a local child FI rate at 22.2%. Within San Antonio, roughly 20% of the population lives below the official poverty line [22]. The SAFB serves over 100,000 clients per week across 29 counties in Southwest Texas.

At the SAFB, research assistants (RAs) spoke to clients waiting for two types of services: Workforce Solutions, which supports employment opportunities/training, and/or Alamo Neighbor services, which helps neighbors access government benefits and other support. Waiting time for these services averages around 30–50 min; therefore, RAs did not disrupt the flow of services at the SAFB and participants were able to complete measures while awaiting services. In an effort to avoid interfering with food distribution services, we purposefully recruited participants who were not waiting to collect food; these services, however, are not mutually exclusive at the SAFB. While recruiting SAFB neighbors, RAs used standardized scripts (both in English and Spanish to depending on the neighbor’s preferred language) to provide a preliminary overview of the study and inquire about interest in study participation. Neighbors who expressed interest received additional information and completed the informed consent prior to enrollment. Of note, secondary to literacy rates among SAFB neighbors, potential participants had the option to read this form independently or for an RA to read the consent form aloud. This was determined by SAFB neighbor self-reported preferences.

After receiving informed consent, all participants received a self-report questionnaire packet. Materials were modified to align with a sixth grade reading level to accommodate the known lower education levels and literacy rates among individuals living with FI. Measures were available in both English and Spanish. Spanish language translation and back translation was completed by bilingual study team members. Subsequently, a San Antonio native reviewed all translations for any remaining cultural or contextual nuanced language adaptions to reflect the local dialect (for a detailed description on the modifications made to the measures for reading level and Spanish language, see Becker et al. 2017 [23]). Spanish-English bilingual RAs were on site at all times for data collection. Participants completed the survey either on a tablet or on paper.

If a participant experienced difficulty reading the survey independently, RAs provided assistance by reading survey items and associated response options aloud. Survey completion time averaged 25 min. All surveys were numerically coded prior to data collection in order to organize the data while maintaining anonymity. As an additional consideration for maintaining confidentiality, participants were asked not to include any identifying information (i.e., names) on the survey. While waiting for in-person services on site, participants completed measures of sociodemographic characteristics, FI, depression, anxiety, QOL, ED symptoms, and eating-related psychosocial impairment. FI and ED symptoms were evaluated via questionnaire (see measures). Although it was generally assumed that individuals receiving services at the SAFB were experiencing food insecurity, this status was confirmed during the assessment. After completing the survey, participants received an $8 gift card to a large grocery store chain in the area. Past studies conducted in partnership with the SAFB have demonstrated that gift cards to the local grocery store chain were preferred by neighbors as compensation for research participation. RAs then debriefed participants and offered a list of local low/no cost mental health services.

### 2.3. Measures

#### 2.3.1. Sociodemographic Information

Sociodemographic characteristics captured by self-report included: gender, age, race and ethnicity, primary language spoken, annual household income, highest level of education, marital status, employment status, number of people and children in the home, disability status, and medical history. Since low-income individuals facing FI and multiple NMDoH barriers do not have consistent access to healthcare, and body weight scales are considered luxury items for households that cannot afford basic needs, we did not inquire about height and weight as this would not capture accurate data.

#### 2.3.2. Food Insecurity (FI)

Radimer/Cornell Food Insecurity Measure (RCFIM; [24,25]). We utilized the 13- item RCFIM to assess severity of FI. This measure uses a 3-point Likert scale to assess food insecurity, where higher scores indicate more severe levels of FI. One item in this measure states, “I know my child(ren) are hungry sometimes, but I can’t afford more food”. The RCFIM classifies respondents into one of the following categories: (1) food secure; (2) household FI (i.e., food scarcity, lack of food variety due to limited resources, anxiety about food,); (3) individual FI (i.e., the respondent experiences hunger because due to lack of food secondary to insufficient resources); (4) child-hunger household FI (i.e., adult respondent reports an inability to feed children due to insufficient resources and that one or more children in the home experience hunger and) (21). Child hunger represents the most extreme form of FI, as adults in the home typically conserve any food to provide for children in their care. As a result, adults in the household are often more affected by hunger than children. Since all research participants were using resources at the SAFB, we reclassified the least severe group as “not food insecure” (NFI) instead of “food secure,” indicating that they are living with insufficient resources in the home leading to some degree of FI. The RCFIM demonstrated strong internal consistency in our present sample (Cronbach’s α = 0.92).

#### 2.3.3. Depression

Personal Health Questionnaire Depression Scale (PHQ-8; [26]). To assess depressive symptoms, we employed the 8-item version of the PHQ. Participants were asked to score how frequently issues like depression and hopelessness bothered them. Responses were recorded on a 4-point Likert scale, and higher scores indicate greater depressive symptoms. We opted to use the PHQ-8 instead of the PHQ-9 because it does not include the item on suicidal ideation (“Thoughts that you would be better off dead, or of hurting yourself”). Research suggests that the PHQ-8 is a valid and reliable measure of depression (Cronbach’s α = 0.88). Both the PHQ-8 and PHQ-9 show a very high correlation (r = 0.997) and comparable sensitivity [27]. Within our sample, the PHQ-8 demonstrated excellent internal consistency (α = 0.93).

#### 2.3.4. Anxiety

Generalized Anxiety Disorder-7 (GAD-7; [28]). We evaluated anxiety symptoms over the past two weeks using the 7-item GAD-7. A 4-point Likert scale is used to rate responses; greater scores reflect greater severity of anxiety symptoms. The GAD-7 is a highly validated and dependable tool for assessing anxiety (α = 0.92). Our sample’s internal consistency was excellent (α = 0.94).

#### 2.3.5. Health Related Quality of Life (HRQOL)

EUROHIS-QOL-8 [29]. We used the 8-item EUROHIS-QOL-8 to measure HRQOL. The World Health Organization Quality of Life Scale (WHOQOL-BREF) served as the model for this short assessment. In order to reduce participant burden, we chose this measure to quantify HRQOL. This scale includes items related to overall quality of life, energy, general health, self-esteem, daily activities, finances, living conditions, and social relationships over the previous two weeks. A 5-point Likert scale was used to rate the items; higher scores corresponded to better HRQOL. The current sample displayed good internal consistency (α = 0.87).

#### 2.3.6. Eating Disorder Symptoms

Eating Disorder-15 (ED-15; [30]). We used the 15-item ED-15 to capture current ED symptoms and the frequency of any ED symptoms over the past week. Items are scored on a 7-point Likert scale, with higher scores indicating greater ED pathology. Two subscales measuring eating and weight/shape concerns make up this measure. Eating attitudes are measured via the ED-15’s first ten items (example item: “Over the past week, how often have I worried about losing control over my eating”). Only those who have engaged in dysregulated behaviors (e.g., binge eating or self-induced vomiting) are asked to respond to the final five open-ended questions. Good clinical validity and reliability has been shown by the ED-15 (current sample α = 0.96; [30]).

#### 2.3.7. Eating-Related Psychosocial Impairment

Clinical Impairment Assessment Questionnaire (CIAQ; [31]). The 16-item CIAQ was used to measure psychosocial impairment due to food and weight-related concerns over the previous month. This measure evaluates how several aspects of life (e.g., cognitive functioning, social engagement, work performance) are affected by exercise, eating habits, and attitudes toward weight and body shape. All responses are recorded on a 4-point Likert scale; higher scores indicate worsened impairment. Overall, the internal consistency and construct validity of the CIAQ are very good [31]; the current sample’s internal consistency was excellent (α = 0.98).

### 2.4. Analytic Strategy

Sociodemographic variability was examined using descriptive statistics, including FI severity, age, and race/ethnicity; bivariate Spearman’s rho correlations examined associations between FI severity and psychosocial variables for the total sample. The primary aim of this study was to investigate potential differences in psychosocial health among women in different life stages all living with FI. We divided participants into four ages groups anchored on developmental life stages (e.g., [32,33]). Although, as noted above, midlife is often considered to start at age 40, recent research has shown that the health conditions that are associated with midlife start as young as 35 [34,35]. Thus, we chose to mirror the age groups used by Miller et al. (2020) in their recent investigation of the association between health conditions and midlife in an adult food insecure sample [4]: (1) young adult (age 18–34 years); (2) early midlife (age 35–49 years); (3) late midlife (age 50–64); and (4) older adult (age 65+ years). This grouping strategy produced fairly equivalent group sizes: young adult (*n* = 162; Mage = 27.0, SD = 4.9), early midlife (*n* = 177; Mage = 42.5, SD = 4.5), late midlife (*n* = 192; Mage = 56.9, SD = 4.4), older adult (*n* = 114; Mage = 70.7, SD = 5.5; upper bound age = 94 years). Scores for all psychosocial measures (e.g., PHQ-8, GAD-7, ED-15, CIAQ) were computed in accordance with standard scoring protocols for each measure. See Table 2 for descriptives by age group and for the total sample.

To examine potential between-group differences in psychosocial health indices while accounting for multiple dependent variables, we conducted a multivariate analysis of covariance (MANCOVA), covarying for known correlates of psychosocial health: FI severity and participant race/ethnicity. Levene’s test of equality of error variances examined the error variance of the dependent variable across groups. We used Pillai’s Trace to assess the variance–covariance matrices and account for unequal matrices when evaluating assumptions because this is a more robust test of true between-groups differences. Because we hypothesized that women living with FI in both midlife stages (early and late midlife) would report worse psychosocial health than the younger and older groups (i.e., a quadratic trend), we conducted planned polynomial contrasts to examine linear, quadratic, and cubic (i.e., k-1 contrasts).

## 3. Results

### 3.1. Total Sample

Overall, 83% of the sample reported living with individual-level or child hunger in the home. Thus, most women in this sample experienced hunger in the home due to insufficient resources. Within the total sample, severity of FI was significantly correlated with depressive symptoms (r = 0.31, *p* < 0.001), anxiety symptoms (r = 0.33, *p* < 0.001), poorer HRQOL (r = −0.34, *p* < 0.001), ED symptoms (r = 0.28, *p* < 0.001), and eating-related psychosocial impairment (r = 0.29, *p* < 0.001).

### 3.2. Omnibus Results

Regarding the primary aim of this study investigating psychosocial health across women in different developmental life stages living with FI, we examined data distribution of variances and variance-covariance matrices prior to interpreting inferential outcomes. Levene’s test of equality of error variances indicated that all dependent variables were of equal variances across groups, except the CIAQ, which measures eating-related psychosocial impairment. Pillai’s Trace was significant for our independent variable (*p* < 0.001), suggesting a true difference between the age groups.

Omnibus results from the MANCOVA indicated significant differences across age groups for anxiety (*p* = 0.001), HRQOL (*p* = 0.043), ED symptoms (*p* = 0.004), and eating-related psychosocial impairment (*p* < 0.001) when covarying for FI severity and race/ethnicity. There was no omnibus age group effect for depressive symptoms. Regarding covariates, FI severity was significantly related to all dependent variables, while race/ethnicity was related to depression, anxiety symptoms, ED symptoms, and eating-related psychosocial impairment. See Table 3 for omnibus MANCOVA results.

### 3.3. Planned Contrasts

Planned polynomial comparisons indicated significant quadratic trends for anxiety symptoms (*p* = 0.005, 95% CI [−2.40, −0.44]), HRQOL (*p* = 0.049, 95% CI [0.004, 1.85]), ED symptoms (*p* = 0.002, 95% CI [−6.46, −1.47]), and eating-related psychosocial impairment (*p* < 0.001, 95% CI [−5.59, −1.48]). The quadratic trend was nonsignificant for depression (*p* = 0.067, 95% CI [ −2.02, 0.07]). Linear trends were significant for anxiety symptoms (*p* = 0.004, 95% CI [ −2.57, −0.49]), ED symptoms (*p* = 0.023, 95% CI [−5.71, −0.42]), and eating-related psychosocial impairment (*p* = 0.008, 95% CI [−5.13, −0.78]); however, inspection of descriptives (means and standard deviations) suggested that this linear trend was primarily influenced by a strong quadratic pattern with lower scores in the older adult group. Thus, the significance of these linear trends is better accounted for by the quadratic between-groups estimates. No cubic trends were significant.

### 3.4. Post Hoc Pairwise Comparisons

Based on planned contrast findings, we conducted post hoc pairwise comparisons using Bonferroni corrections for multiple tests to further investigate potential differences in psychosocial health across the lifespan and enhance the depth of interpretation into the nature of group differences (e.g., potential differences between early and late midlife, or between late midlife and older adult women). No post hoc pairwise comparisons were significant for depression after adjusting for multiple comparisons. For anxiety, the older adult group (age 65+) endorsed significantly lower GAD-7 scores than the early-midlife (age 35–49). All other post hoc multiple pairwise comparisons significantly differed between age groups. For anxiety, the older adult group endorsed significantly lower GAD-7 scores than all three other age groups: (1) versus the young adult group (*p* = 0.01, 95% CI [−4.41, −0.40]); (2) versus the early midlife group (*p* = 0.008, 95% CI [−4.44, −0.43]); and (3) versus the late midlife group (*p* < 0.001, 95% CI [−4.75, −0.86]). No other pairwise comparisons were significant for anxiety symptoms. Regarding HRQOL, only the young adult and late midlife groups significantly differed (*p* = 0.03, 95% CI [0.10, 3.35]), with the young adult group endorsing better HRQOL than the late midlife group. The older adult group endorsed significantly lower ED symptoms than the early midlife group (*p* = 0.002, 95% CI [−12.07, −1.90]). Finally, the older adult group endorsed significantly lower eating-related impairment than the early midlife (*p*, 0.001, 95% CI [−10.66, −2.30]) and late midlife (*p* = 0.034, 95% CI [−8.32, −0.21]) groups; no other pairwise comparisons were significant.

## 4. Discussion

The primary aim of the current study was to investigate mental health and HRQOL across developmental life stages among women living with FI. Research supports a link between mental health concerns and suboptimal nutrition, including reduced intake of important micronutrients [36,37]. Importantly, living with FI represents a chronic state of suboptimal nutritional intake both comprising overall malnutrition and/or elevated intake of macronutrient-dense, highly processed foods, and lower access to micronutrient dense foods. Although FI is a well-established risk factor for mental health concerns, the impact of living with FI among women during different life stages remains largely understudied. This is especially the case when reviewing the research that has engaged in-person samples living with FI versus nationally representative datasets which included a subsample endorsing FI (e.g., [21,38,39]). This study was conducted in partnership with the SAFB, on site, with neighbors presenting for services.

This sample was living with significant food insecurity; the majority of this sample (83%) reported living with individual or child hunger in the home, indicating that adults and children in the home experience hunger. Consistent with substantial prior literature, higher FI severity was significantly correlated with poorer mental health and HRQOL among women (age range 18–94) living with FI and presenting to a food bank for services.

Our hypotheses were partially supported; anxiety, HRQOL, ED symptoms, and eating-related psychosocial impairment significantly differed across developmental age group among women living with FI. There were no differences in depressive symptoms by age group. FI severity was significantly related to all outcomes, which was reflected in bivariate correlations in the total sample. Yet, women’s developmental age group differences emerged for anxiety, HRQOL, ED symptoms, and eating-related impairment even after covarying for this effect of FI severity as well as race/ethnicity. Specifically, significant quadratic trends indicated worse anxiety, HRQOL, ED symptoms, and eating-related psychosocial impairment, when covarying for FI severity and race/ethnicity, among the two midlife groups (i.e., early and late midlife (respective ages 35–49 and 50–64)) as compared to younger (age 18–34) and older (age 65+) groups. Similarly, post hoc pairwise comparisons further supported this interpretation.

Although living with FI is an established risk factor for poorer mental health, a deeper understanding into how women in different developmental life stages—and therefore facing qualitatively different social, psychological, and physical factors secondary to the aging process—are impacted by living with FI. The majority of our sample was of Hispanic/Latina ethnicity, a culture in which multigenerational homes are common [40]. For women in midlife, this often means facing caregiving roles both of minors in the home and of older adult family members resulting in increased toll on mental health. Additionally, the sense of responsibility as a multigenerational caregiver in the face of insufficient food access for either generation can amplify anxiety or distress. Women in midlife may prioritize feeding children and older adults in the home in line with caregiving roles, thus eliciting further risk for hunger and its mental health toll. Importantly, past research in non-US samples found that FI mediates the relation between poverty and psychological distress [41]. Additionally, midlife may be a particularly vulnerable period for the development of disordered eating. The chronic, unpredictable nature of FI can lead to cyclical patterns of restriction and overeating, which closely resemble those seen in clinical eating disorders. This stage of life is also marked by the menopausal transition, a period often characterized by changes in body image/identity, along with hormonal shifts and weight fluctuations [42,43]. For midlife women already navigating financial strain and caregiving responsibilities, these intersecting stressors may further elevate the risk for eating-related impairment.

Finally, it is important to recognize that the relationship between FI and mental health may be bidirectional. Psychopathology, such as anxiety, may impair an individual’s ability to maintain stable employment and housing, thereby increasing vulnerability to FI [44]. Similarly, the chronic stress associated with living with FI may worsen pre-existing mental health conditions [45]. Understanding this relationship is essential for developing interventions that address both the psychological and physical needs of individuals experiencing FI.

### Limitations

Limitations of the current study include the use of self-report data for psychosocial constructs and its cross-sectional design, which limits our ability to investigate mediators or capture chronological risk related to mental health for women in different developmental life stages. Longitudinal designs would allow us to investigate chronicity of mental health concerns and FI; it could be the case that women with poorer mental health are more likely to experience FI and have more difficulty coping with the demands of multigenerational caregiving. On the other hand, living with a prolonged suboptimal nutritional state has known effects on the human body, including cognition, emotional health, and physical health [41]. For instance, past research suggests that the transactional impact of stress and poor nutritional status among individuals living with FI results in exponentially increased allostatic load (e.g., [46,47]) causing a cascade of poorer health outcomes [48] and further exacerbating health disparities in populations living with FI. Thus, longitudinal studies will be imperative in detecting mechanisms of symptom risk, onset, and maintenance, and in identifying pathways for intervention. Additionally, while our age groups had unequal sample sizes, the ratio of groups did not approach 1:2, the use of multivariate analyses with covariate adjustments reduced the likelihood of unequal sample size influence, Pillai’s Trace analyses indicated that true differences between groups existed, and Levine’s Tests supported homogeneity of variances. To further remedy concerns related to unequal sample sizes, future research should selectively sample women of different ages living with FI. An additional limitation is that participants were recruited from neighbors who were seeking assistance with benefits and employment versus those who sought food, and it is possible that these samples could differ fundamentally in some way. However, it is worth noting that overall rates of FI and financial status did not substantially differ between this study and results from Becker et al. [23], which recruited participants who were seeking food from food pantry partners of the SAFB. In the current sample, 94.5% of respondents were categorized as food insecure, while 91.8% of the sample in the Becker et al. paper recruited from food pantries (i.e., seeking food assistance at that time of participation) were categorized as food insecure. Finally, the literature defining the upper and lower limits for “midlife” among women is inconsistent. Despite these inconsistencies, we used upper and lower bound age parameters consistent with literature in FI populations that distinguish earlier and later midlife periods in women’s developmental lifespan [4]. Lastly, sociodemographic factors were not included as covariates in our analyses, though they may confound the relationship between FI and mental/physical health outcomes. Future research should incorporate these variables to better understand how this relationship changes over the lifespan.

## 5. Conclusions

Findings from the current study augments a growing body of literature demonstrating an association between living with FI and poorer mental health among women [21], while extending our understanding into potential age-related exacerbations of this association for women across the lifespan. Women in early and late midlife reported a greater mental health toll of living with FI, even controlling for severity of FI, thus highlighting a developmental life stage-related window of vulnerability for experiencing the mental health burden of living with FI. Results from this study offer early evidence in support of future policy and advocacy work toward supporting women in midlife living with FI and other NMDoH barriers to health, with an enhanced emphasis on supporting women in midlife (i.e., the “sandwich generation”) via intervention, prevention, and overall resource allocation. Increase advocacy and awareness efforts are needed, in order to increase screening for the intersection of FI and mental health concerns in healthcare settings that serve women, but through a lens of developmental life stage considerations in the context of FI and mental health. For example, women in midlife and older are not routinely asked about disordered eating behaviors; yet, our data show that women in early and late midlife with FI endorsed more ED symptoms than younger women, thus challenging the ED stereotype as disorders of youth.

Efforts are needed to develop and/or delineate how systems-level approaches could be leveraged to support women in midlife living with FI (vs. the traditional mental healthcare model of 1:1 therapy and ownness for change on the individual, which assumes resources are available for individual level change). We will likely need a restructuring of mental health support systems and processes if we are to fully respond to these mental health needs. Specifically, routine screening for FI and mental health should be incorporated into primary care and community health settings, particularly for midlife and older women who are often overlooked. Food banks and community organizations could partner with mental health providers to offer accessible, co-located behavioral health services and support groups for vulnerable populations. At a broader level, collaboration across healthcare, housing, employment, and social services is essential to address complex challenges faced by women living with FI. Interventional and resource pathways will likely include options for multi-level and/or multicomponent intervention strategies to support women living with FI and make strides to improve health equity for this population.

## Figures and Tables

**Table 1:** Participant demographics summarized for the total sample and by age group.

Demographic	Total Sample *N* = 680		Young Adult (18–34) *n* = 176		Early Midlife (35–49) *n* = 183		Late Midlife (50–64) *n* = 201		Older Adult (65+) *n* = 120	
	*n*	*%*	*n*	*%*	*n*	%	*n*	%	*n*	%
Ethnicity										
Latino/Hispanic	540	79.4	134	76.1	151	82.5	159	79.1	96	80
Black/African American	31	4.6	11	6.3	8	4.4	8	4	4	3.3
White/Caucasian	63	9.3	14	8.0	10	5.5	27	13.4	12	10
Other ^+^	46	6.7	14	9.7	14	7.5	7	3.5	8	6.6
Food Insecurity										
Not food insecure	36	5.3	9	5.1	10	5.5	8	4	9	7.5
Household FI	78	11.5	31	17.6	20	10.9	11	5.5	16	13.3
Individual FI	287	42.2	55	31.3	43	23.5	113	56.2	76	63.3
Child Hunger	279	41	81	46	110	60.1	69	34.3	120	15.8
Education										
No or some grade school	61	9	11	6.3	11	6	14	7	25	20.8
Finished grade school	32	4.7	5	2.8	5	2.7	12	6	10	8.3
Some high school	134	19.7	21	11.9	48	26.2	47	23.4	18	15
High school/GED	253	37.2	92	52.3	61	33.3	70	34.8	30	25
Some college or technical	154	22.6	33	18.8	48	26.2	46	22.9	27	22.5
Bachelor ^+^	46	6.8	14	7.9	10	5.4	12	6	10	8.3
Annual Household Income										
<$10,000	318	49.4	93	57.1	83	46.9	86	44.6	56	50.5
$10,000–$40,000	295	43.4	61	37.5	84	47.5	99	51.3	51	45.9
$40,000–$65,000	24	3.7	7	4.3	9	5.1	4	2	4	3.6
$65,000+	7	1.1	2	1.2	1	0.5	4	2		
Children in the Household										
0	299	44.4	43	24.4	39	21.4	125	62.8	92	78
1	140	20.7	46	26.1	46	25.3	35	17.6	13	11
2	111	16.4	33	18.8	52	28.6	19	9.5	7	5.9
3	66	9.8	26	14.8	22	12.1	13	6.5	5	4.2
4 or more	59	8.7	28	15.9	23	12.5	7	3.5	1	0.8
Employment Status										
Disabled	140	20.6	8	4.5	27	14.8	68	33.8	37	30.8

Notes. ^+^ Some racial groups were collapsed into ‘other’ due to small sample sizes, including Asian, Native American, Pacific Islander, Indigenous/Aboriginal, and multiple races.

**Table 2:** Descriptive Statistics for Dependent Variables Separated by Age Group.

Measures	Total *M* (SD)	Young Adult *M* (SD)	Early Midlife *M* (SD)	Late Midlife *M* (SD)	Older Adult *M* (SD)
Depression (PHQ-8)	8.7 (6.8)	8.8 (6.8)	9.8 (7.1)	8.8 (6.7)	6.8 (6.3)
Clinical Cutoff (%)		36.4%	43.7%	38.8%	25.8%
Anxiety (GAD-7)	8.9 (6.5)	9.2 (6.4)	9.6 (6.8)	9.7 (6.7)	6.1 (5.3)
Clinical Cutoff (%)		40.9%	44.8%	46.8%	22.5%
Quality of Life	25.0 (6.2)	26.0 (6.3)	24.3 (6.5)	24.3 (6.0)	25.8 (5.5)
Eating Disorder Symptoms	14.7 (16.2)	14.3 (15.8)	17.9 (17.5)	15.3 (16.3)	9.2 (13.2)
Clinical Impairment (CIAQ)	10.8 (13.4)	10.6 (12.7)	13.7 (14.8)	11.2 (13.9)	6.0 (9.1)
Clinical Cutoff (%)		27.3%	34.4%	31.3%	11.7%

**Table 3:** MANCOVA Omnibus Results.

		Measures	F	Sig.	Partial Eta Squared
*Covariates*	FI Severity	Depression	46.192	<0.001	0.067
		Anxiety	51.836	<0.001	0.075
		Quality of Life	87.142	<0.001	0.120
		Eating Disorder Symptoms	23.667	<0.001	0.036
		Clinical Impairment	20.333	<0.001	0.031
	Ethnicity	Depression	4.317	0.038	0.007
		Anxiety	6.014	0.014	0.009
		Quality of Life	1.226	0.269	0.002
		Eating Disorder Symptoms	8.903	0.003	0.014
		Clinical Impairment	10.397	0.001	0.016
*Fixed Factor*	Age Group	Depression	2.095	0.100	0.010
		Anxiety	5.407	0.001	0.025
		Quality of Life	2.736	0.043	0.013
		Eating Disorder Symptoms	4.510	0.004	0.021
		Clinical Impairment	5.676	<0.001	0.026

Note: Effect size interpretation: Small = 0.01; Medium = 0.06; Large = 0.14.

## Data Availability

The dataset generated and/or analyzed during the current study is not publicly available but is available from the corresponding author upon reasonable request.

## Abbreviations

The following abbreviations are used in this manuscript:

US	United States of America
ED	Eating Disorder
ED-15	15-Item Eating Disorder Pathology Questionnaire
FI	Food Insecurity
RAs	Research Assistants
IRB	Institutional Review Board
HRQOL	Health-related Quality of Life
RCFIM	Radimer/Cornell Food Insecurity Measure
GAD-7	7-Item Generalized Anxiety Disorder Questionnaire
PHQ-8	8-Item Personal Health Questionnaire
EUROHIS-QOL-8	8-Item European Health Interview Survey-Quality of Life
CIAQ	Clinical Impairment Assessment Questionnaire
CI	Confidence Interval
SAFB	San Antonio Food Bank
MANCOVA	Multivariate Analysis of Covariance
NMDoH	Nonmedical Determinants of Health
